# Metastatic pancreatic adenocarcinoma presenting as large bowel obstruction: A case report

**DOI:** 10.1016/j.ijscr.2022.107801

**Published:** 2022-11-23

**Authors:** Felipe Pacheco, Emmanuel Luciano, Danielle Hebert, Omar Marar

**Affiliations:** Department of Surgery, Central Michigan University College of Medicine, Saginaw, MI, United States

**Keywords:** Case report, Metastatic pancreatic cancer, Large bowel obstruction

## Abstract

**Introduction and importance:**

The incidence of pancreatic cancer has gradually increased over the past decades. Metastatic pancreatic cancer to the colon is rare with only seven cases reported. Symptomatic metastasis to the colon as the initial presentation of pancreatic adenocarcinoma has only been reported in two occasions prior to this report.

**Case presentation:**

In this report, the diagnosis and operative management of a 78-year-old male who presented with three days of obstipation and computed tomography evidence of an annular obstructing mass in the sigmoid colon in addition to a pancreatic body and lesser sac mass involving the gastric antral region. The patient underwent a laparoscopic sigmoidectomy with end colostomy. Pathology revealed metastatic adenocarcinoma of pancreatic origin. The postoperative period was unremarkable, and the patient was referred to medical oncology to pursue further treatment.

**Clinical discussion:**

Symptomatic metastasis to the colon as the initial presentation of pancreatic adenocarcinoma is exceedingly rare. To our knowledge, this is the 3rd case to be reported and the second that was located in the sigmoid colon.

**Conclusion:**

The presentation of metastatic pancreatic cancer to the colon causing a colonic obstruction is rare but should be considered in cases of atypical synchronic masses in cross-sectional imaging. When metastatic disease is suspected in the sigmoid colon, it is advisable to perform a colostomy instead of an anastomosis to avoid the potential risk of anastomotic leak that could delay the immediate need for systemic therapy.

## Highlights

• Pancreatic cancer metastatic to the colon is rare.
• 3rd case of symptomatic metastasis to the sigmoid colon as the initial presentation of pancreatic cancer
• Complete colonic obstruction required emergent surgical intervention.

## 1 Introduction and importance

The incidence of pancreatic cancer has gradually increased over the past decades, currently being the 14th most common cancer and the 7th highest cause of cancer related mortality worldwide [1]. Pancreatic cancer is usually diagnosed in advanced stages due to the asymptomatic nature in the early stages of the disease. About 50 % of patients with pancreatic cancer have metastatic disease at the time of presentation, most commonly in the loco-regional lymph nodes, liver, and lungs [2]. Metastatic pancreatic cancer to the colon is rare with only seven cases reported [4], [5], [6], [7], [8], [9], [10]. Symptomatic metastasis to the colon as the initial presentation of pancreatic adenocarcinoma has only been reported in two occasions prior to this report [4], [9]. In this manuscript, the diagnosis and operative management of a 78-year-old male who presented with three days of obstipation and cross-sectional imaging evidence of complete large bowel obstruction due to a sigmoid mass, in addition to an incidental pancreatic mass is described. This work has been reported in line with the SCARE 2020 criteria [11].

## 2 Case presentation

**Image 1:**
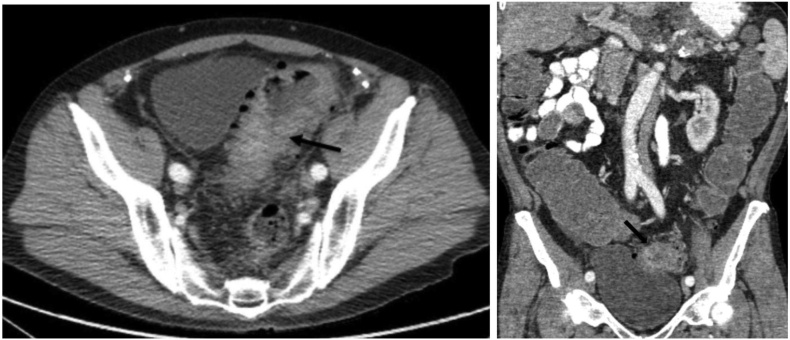
Axial (left) and coronal (right) CT scan - sigmoid mass (black arrow).

**Image 2:**
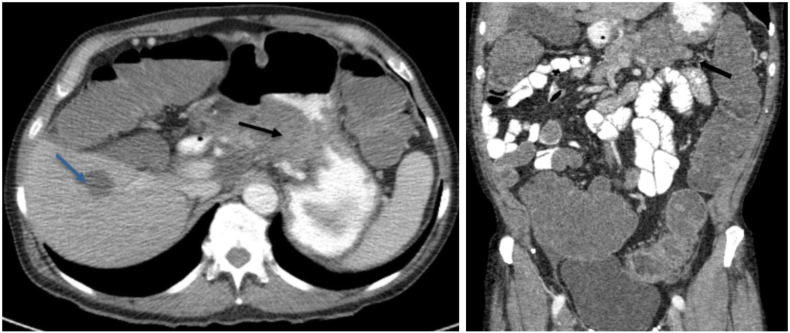
Axial (left) and coronal (right) CT scan - large pancreatic body mass invading the posterior wall of the stomach (black arrow) and hepatic metastatic lesion (blue Arrow).

A 78-year-old male with a past medical history of bladder cancer in remission following endoscopic treatment, noncontributory psychiatry and social history, and a normal colonoscopy four years prior to presentation. The patient presented to the emergency department (ED) with a four-day history of diffuse abdominal pain, distention, nausea and one episode of bilious emesis. His last bowel movement was three days prior to presentation. The abdomen was distended with diffuse tenderness and no signs of peritonitis. Initial laboratory workup was unremarkable. A computed tomography (CT) of the abdomen and pelvis showed an annular obstructing mass in the sigmoid colon of 4.8 cm in length in addition to a pancreatic body and lesser sac mass involving the gastric antral region and a hepatic lesion suspicious for metastatic disease (Image 1, Image 2). The cancer antigen (CA) 19–9 level was elevated to 112,444 U/ml (normal <35 U/ml).

Due to the complete colonic obstruction, urgent surgical resection was deemed necessary. Preoperatively, a nasogastric tube was placed, and the patient was resuscitated with isotonic fluids. The procedure was performed by the senior colorectal surgeon in an academic hospital. A laparoscopic approach was chosen. A superficial liver lesion in the segment 4B was identified and a biopsy was performed using laparoscopic biopsy forceps. The abdomen was inspected and no evidence of carcinomatosis was found. On exploration of the pelvis, an obstructing mass in the sigmoid was visualized with decompressed distal rectum. A sigmoidectomy was performed utilizing a laparoscopic gastrointestinal anastomosis (GIA) stapler. A high ligation of the inferior mesenteric artery (IMA) was performed for oncologic purposes. Due to the anticipated need for urgent chemotherapy the decision to defer an anastomosis was made and an end-colostomy was created.

The postoperative course was unremarkable. The patient had return of bowel function on postoperative day (POD) 1, the diet was advanced as tolerated and he was discharged on POD 3. The patient was seen in the office 2 weeks after his surgery with no major complaints. Pathology for the sigmoid specimen was consistent with 5 out of 10 lymph nodes positive for metastatic disease. Likewise, the liver biopsy specimen was consistent with the same findings. The patient was referred to medical oncology for further systemic therapy.

## 3 Clinical discussion

Metastatic pancreatic cancer is a fairly common diagnosis in the western world with an uprising incidence. It frequently metastasizes to regional or distant lymph nodes, liver, lung, and peritoneum [3]. Metastatic pancreatic cancer to the colon is rare with only seven cases reported prior to our case [4], [5], [6], [7], [8], [9], [10]. Four cases presented in the acute setting with colonic obstruction requiring surgical exploration. In three of these cases the metastatic obstructing area was located in the ascending colon and underwent right hemicolectomy. The remaining case was located in the sigmoid and underwent sigmoidectomy with end colostomy. Two of these cases were in patients with undiagnosed pancreatic cancer [4], [5], [6], [9].

Kelly et al. reported the first case of symptomatic metastasis to the sigmoid colon as the initial presentation of pancreatic cancer, and similar to our case, underwent sigmoidectomy with end colostomy creation [4]. As we anticipated the need for urgent systemic chemotherapy, we elected to create an end colostomy instead of an anastomosis in order to avoid the consequences and delay in systemic treatments due to potential anastomotic related complications.

## 4 Conclusion

Patients with completely obstructing colon tumors are a surgical emergency, usually requiring segmental colonic resection. The presentation of metastatic pancreatic cancer to the colon causing a colonic obstruction is rare but should be considered in cases of atypical synchronic masses in cross-sectional imaging. Finally, when metastatic disease is suspected in the sigmoid colon, it is advisable to perform a colostomy instead of an anastomosis to avoid the potential risk of anastomotic leak that could delay the immediate need for systemic therapy.

## Consent

Written informed consent was obtained from the patient for publication of this case report and accompanying images. A copy of the written consent is available for review by the Editor-in-Chief of this journal on request.

## Sources of funding

No external funding was available for this study.

## Ethical approval

None required.

## Research registration

N/a.

## Guarantor

Felipe Pacheco, MD.

## Provenance and peer review

Not commissioned, externally peer-reviewed.

## Credit authorship contribution statement

Felipe Pacheco, MD: conceptualization, methodology, writing original draft and final review and editing.

Emmanuel Luciano, MD: writing original draft and final review and editing.

Danielle Hebert, MD: writing original draft.

Omar Marar, MD: final review and editing.

## Declaration of competing interest

None declared.
